# SenGlove—A Modular Wearable Device to Measure Kinematic Parameters of The Human Hand

**DOI:** 10.3390/bioengineering10030324

**Published:** 2023-03-03

**Authors:** Jonas Paul David, Thomas Helbig, Hartmut Witte

**Affiliations:** 1 Fachgebiet Biomechatronik, Institut für Mechatronische Systemintegration, Fakultät für Maschinenbau, Technische Universität Ilmenau, 98693 Ilmenau, Germanythomas.helbig@tu-ilmenau.de (T.H.); 2 neuroConn GmbH, Albert-Einstein-Straße 3, 98693 Ilmenau, Germany

**Keywords:** wearable devices, wearable sensors, data glove, biomechatronic design, biomedical engineering, hand kinematics, joint measurement, flex sensors

## Abstract

For technical or medical applications, the knowledge of the exact kinematics of the human hand is key to utilizing its capability of handling and manipulating objects and communicating with other humans or machines. The optimal relationship between the number of measurement parameters, measurement accuracy, as well as complexity, usability and cost of the measuring systems is hard to find. Biomechanic assumptions, the concepts of a biomechatronic system and the mechatronic design process, as well as commercially available components, are used to develop a sensorized glove. The proposed wearable introduced in this paper can measure 14 of 15 angular values of a simplified hand model. Additionally, five contact pressure values at the fingertips and inertial data of the whole hand with six degrees of freedom are gathered. Due to the modular design and a hand size examination based on anthropometric parameters, the concept of the wearable is applicable to a large variety of hand sizes and adaptable to different use cases. Validations show a combined root-mean-square error of 0.99° to 2.38° for the measurement of all joint angles on one finger, surpassing the human perception threshold and the current state-of-the-art in science and technology for comparable systems.

## 1. Introduction

### 1.1. Overview

The human hand with its opposable thumb is a unique feature of *Homo sapiens* [1,2]. The hand is an essential tool for handling and manipulating all types of objects and also communicating with other humans or machines. To utilize all these features for medical and technical applications, the measurement of the exact kinematics or at least knowledge of certain gestures of the hand with its high degree of freedom (DoF) is gaining increasing interest. Therefore, a rising number of wearable electronics—especially as part of e-textile systems—are being developed with a promising outlook in markets [3]. In the wearable healthcare market alone, a volume of USD 46.6 billion is predicted for the year 2025. Additional fields of application, such as robot control and teleoperation [4,5,6,7], medicine and rehabilitation [8,9,10,11,12,13] or translation of sign language [14,15,16,17,18,19,20] and more (cp. [21]), add to that volume.

### 1.2. Related Work

A broad variety of wearable devices has been developed for the human hand to use [3,21]. Very often, the main focus is on an active, actoric support of the hand with exoskeleton systems [8,9,10,12,13,22], the provision of haptic feedback [7] or non-kinematic parameters such as the measurement of pressure [23,24] or the use of electromyography (EMG) [9,11,25]. Especially for the use of EMG, the wearable has to be extended at least to the lower arm [25]. For the measurement of kinematic parameters, vision-based systems are used very often [20,26,27], but with a focus on mobile body-bound systems, this article does not investigate them further. Non-visual acquisition of kinematic parameters of the hand—especially the angle of the finger joints—is executed using fiber-optic sensors [28], Hall effect sensors [29], inertial measurement units (IMU) [13,30,31,32,33] or bending/flex sensors [34]. To store additional hardware (e.g., actoric components or battery packs), bag packs [9], belts [35,36] or cuffs [7] are used. Furthermore, smart textiles or sensors [37,38] or the use of soft robotic concepts [9,12,22,35,36] are discussed in the literature.

### 1.3. Our Contribution

In this study, we propose a modular sensorized glove (SenGlove) that measures the kinematic parameters of the hand as the main functionality. To reduce the number of measurement parameters, the complexity of the hand is reduced from DoF 23 [39] to DoF 15, applying biomechanical assumptions and concepts. Anthropometric data of the hand are analyzed to secure the applicability of the SenGlove concept to a broad range of individuals, as well as to introduce a possible sizing system. The use of commercially available components is supposed to reduce costs and achieve high proximity to later product development. The concept of a biomechatronic system [40] forms the basis for the design process and helps to achieve a high degree of user acceptance and to measure without interference of the future user. Based on the theory of systems, the concept of a biomechatronic system helps make the complexity of the existing task manageable and assigns explicit tasks to certain system components. Basic concepts of the mechatronic design process [41] are utilized to gain an optimized design and to reach high flexibility of the system in regard to different applications in the future. The model-based system design is used to break down the overall function—here measuring the kinematic of the hand—of the system into subfunctions, including mechanical structure, electrical structure, and information technology. For these subfunctions, domain-specific development is performed to find optimal solutions for each part of the system. All parts are brought together in the following system integration. With this approach, we achieve a high degree of modularization at the same time.

## 2. Materials and Methods

In this section, basic assumptions and requirements for SenGlove, as well as elements of the domain-specific development following VDI 2206 “Design methodology for mechatronic systems” [41], are described. Only the final design is shown in detail. Design variants discussed during the development are shown briefly in Appendix A.

### 2.1. Basic Assumptions and Requirements/Aims of the Design

The proposed wearable is supposed to be used unrestricted and autonomously in daily life activities by both women in the 5th percentile and men in the 95th percentile (size adaption of the concept). Donning and doffing shall be performed by the user themself without assistance. SenGlove is supposed to measure “absolute” angular information of the finger joints (kinematic measurements) in the first place. In regard to the human perception threshold for angular resolution in the finger joints [42], the measurement accuracy of SenGlove is demanded to be below 2.5°. Additional measurement parameters are pressure information at the fingertips (contact pressure measurements), as well as general information on the movement of the hand (inertial measurements). SenGlove is not intended to be used while handling objects. Therefore, measured contact pressure at the fingertips is regarded as finger–finger or finger–palm contact. No visual analysis is used.

### 2.2. Simplified Hand Model

**Figure 1:**
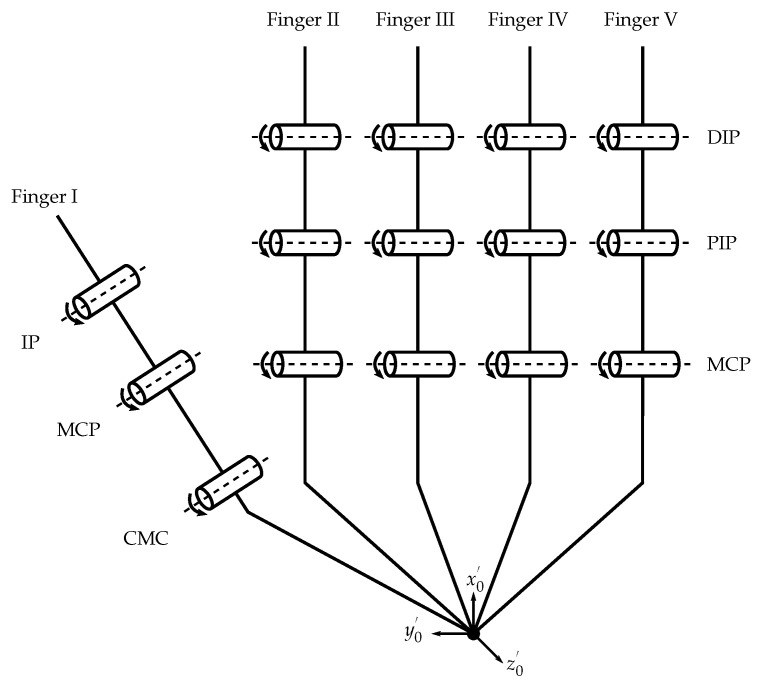
Simplified kinematic modelof a human hand with a degree of freedom of 15. Fingers numbered I (thumb) to V (pinkie finger). Joints: *Carpometacarpal* (CMC), *Distal Interphalangeal* (DIP), *Interphalangeal* (IP), *Metacarpophalangeal* (MCP), *Proximal Interphalangeal* (PIP). Global coordinate system with the axes $x_{0}^{{}^{\prime }}$, $y_{0}^{{}^{\prime }}$ and $z_{0}^{{}^{\prime }}$. In anatomical terms: $x_{0}^{{}^{\prime }}$ points distal, $y_{0}^{{}^{\prime }}$ points radial, $z_{0}^{{}^{\prime }}$ points palmar.

An anatomical model of the human hand has a DoF of 23 [1,39]. In order to take a first prototypical approach of a wearable for measuring the kinematic data of the human hand, a simplified kinematic model of the hand with a DoF of 15 was applied (see Figure 1). Fingers were numbered from thumb (I) to pinkie finger (V) (in anatomy, defined as Digiti DI to DV). To achieve a reduction in DoF, abduction and adduction in the *Metacarpophalangeal* (MCP) joints of each finger and in the *Carpometacarpal* (CMC) joint of the finger I are neglected. The model depicts the finger joints as mechanical hinge joints with a DoF of 1. In addition, the CMC joints of finger IV and V are neglected, as they only allow minimal movement [1,39]. To further reduce the model’s DoF, a constraint based on the anatomy and tendon apparatus of the hand is applied [1]. It describes the coupling between the flexion and extension movements of the *Distal Interphalangeal* (DIP) and *Proximal Interphalangeal* (PIP) joints of the fingers II to V with the linear Equation (1).

(1) $$\theta_{DIP}=\frac{2}{3}\theta_{PIP},$$

where $\theta_{DIP}$ refers to the flexion angle of the DIP joint, and $\theta_{PIP}$ refers to the flexion angle of the PIP joint [43]. This assumption is valid only in free motion of the fingers without additional external forces.

The resulting model is shown in Figure 1. The coordinate system with the axes $x_{0}^{{}^{\prime }}$, $y_{0}^{{}^{\prime }}$ and $z_{0}^{{}^{\prime }}$ was defined as a global coordinate system [43]. It is located in the center of the wrist of the hand model. In addition, each joint axis ji has a local coordinate system $K_{ji}$, where *j* refers to the *j*th finger at which the considered joint is located, and *i* indicates the considered axis of a joint.

### 2.3. Denavit–Hartenberg Method

The coordinate systems $K_{ji}$ follow the rules of the Denavit–Hartenberg convention [43,44]. With the Denavit–Hartenberg method (DH method), it is possible to determine the relative position and orientation of two neighboring coordinate systems within a kinematic chain in three-dimensional space with only four instead of the usual six parameters—e.g., used in [4,13]. The following parameters (DH parameter) are used for the implementation of the DH method [43,44]:

- Joint angle $\theta_{ji}$ of the joint axis ji describes the angle of rotation from the axis $x_{ji-1}$ to the axis $x_{ji}$ around the axis $z_{ji-1}$.
- Joint distance $d_{ji}$ describes the translation of the origin of the coordinate system $K_{ji-1}$ along the axis $z_{ji-1}$ so that the distance between the origins of $K_{ji-1}$ and $K_{ji}$ becomes minimal.
- Link length $a_{ji}$ describes the translation of the origin of the coordinate system $K_{ji-1}$ along the axis $x_{ji}$ so that the distance between the origins of $K_{ji-1}$ and $K_{ji}$ becomes minimal.
- Link twist angle $\alpha_{ji}$ describes the angle of rotation from the axis $z_{ji-1}$ to the axis $z_{ji}$ around the axis $x_{ji}$.

**Figure 2:**
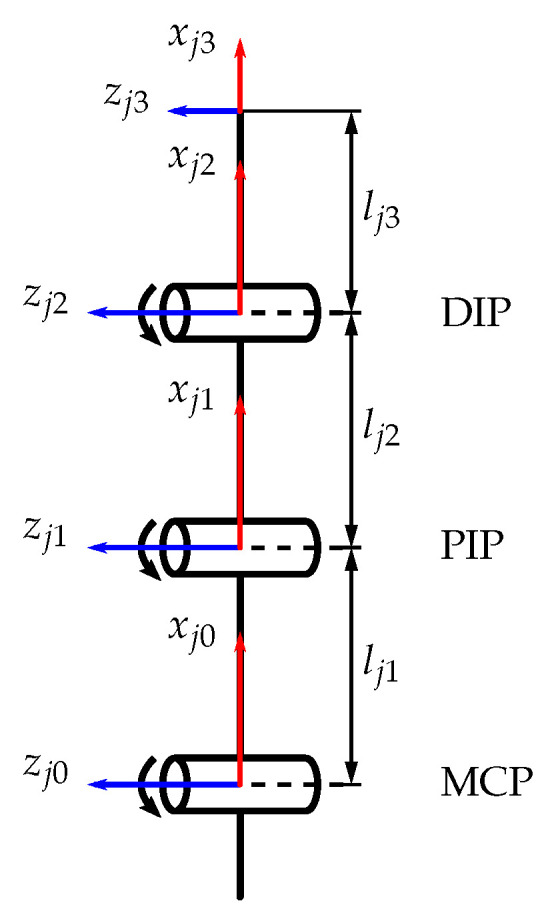
Open kinematic chain of finger II to V (j={2,3,4,5}) with the *Metacarpophalangeal* (MCP), *Proximal Interphalangeal* (PIP) and *Distal Interphalangeal* (DIP) joints. For better distinction, the $x_{ji}$ axes of the local coordinates $K_{ji}$ of each joint are red and the $z_{ji}$ axes are blue. The distances $l_{ji}$ correspond to the functional lengths of the phalanges of the fingers.

Figure 2 shows the open kinematic chain of fingers II to V. The kinematic chain of finger I is analogous to the CMC, MCP and *Interphalangeal* (IP) joints. The DH parameters of the fingers are shown in Appendix C.

In order to transform a local coordinate system $K_{ji-1}$ into the neighboring coordinate system $K_{ji}$, the homogeneous transformation matrix Tji−1ji, also called the Denavit–Hartenberg matrix, from Equation (2) can be applied [44]:

(2) Tji−1ji=cosθji−sinθjicosαjisinθjicosαjiajicosθjisinθjicosθjicosαji−cosθjicosαjiajisinθji0sinαjicosαjidji0001

This homogeneous transformation matrix $T_{Fj}$ describes the position and orientation of the local coordinate system $K_{jn}$ at the tip of the finger *j* with respect to the base coordinate system $K_{j0}$ [44]. The matrix is calculated from the product of all Denavit–Hartenberg matrices of the kinematic chain according to Equation (3):

(3) TFj=Tj0jn=∏i=1nTji−1ji

### 2.4. Hand Size Examination

**Figure 3:**
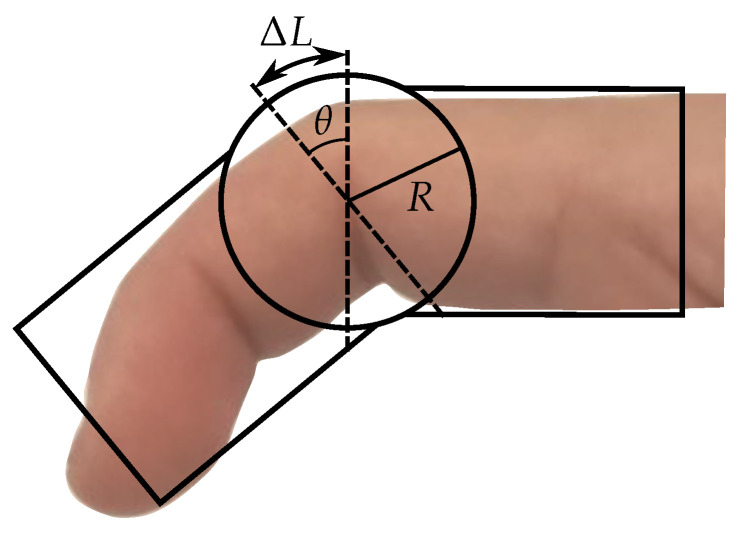
Increase in length ΔL of the skin over the finger joint due to flexion. Depending on the radius of the joint *R* and deflection angle θ.

The anthropometric parameters of the hand, especially the lengths of the fingers, have been examined to lay out the size of SenGlove. Here it must be taken into account that the dorsal skin at the joints stretches when the fingers are flexed [43,45]. The maximum possible length of the fingers and phalanges is relevant for the selection of sensors for the developed sensor concept (see Section 2.6). For the following investigations and estimations, the middle finger is used as a reference, as it is usually the longest finger of the hand [46]. Table A1 contains the values of middle finger lengths for women and men for different percentiles as determined by *Tilley 2002* [46]. The length of the middle finger was measured as the distance from the tip of the finger to the midpoint of the MCP joint, with the finger fully extended. The stretching of the skin when the middle finger is bent results in an increase in the length ΔL of the skin along the longitudinal axis of the finger, which is crucial for the choice and placement of sensors on the hand [43,45]. The anatomy of finger joints shows that the articulating surfaces of a joint have different radii of curvature [47]. Accordingly, the axis of rotation of the finger joints is not a fixed axis but a helical axis [48] that shifts during flexion and extension of the joint. According to Li et al. 2011, these displacements are negligible compared to the dimensions of the finger [43]. *Burfeind 2004* determined maximum displacements of the axes of rotation of 1 mm in the MCP joint, 0.13 mm in the PIP joint, and 0.19 mm in the DIP joint [47]. This allows assuming the axis of the rotation of the finger joints to be stationary and modeling the rotation of the joints by rotating a circular disc of constant radius *R* (see Figure 3).

The increase in length ΔL can be calculated with the deflection angle θ, and the radius *R* of the joint as the arc length according to Equation (4):

(4) $$\Delta L=\frac{2\pi \theta }{360}\;\cdot \;R$$

Following Equation (4), the maximum increase in length for the MCP, PIP and DIP joints of a middle finger is calculated for different percentiles in men and women. For θ, the angular data of maximum flexion according to [39] are used (see Table A2). Radius *R* is derived from anthropometric studies on finger thickness at the location of the joints, according to [46]. Results are given in Table A3.

Adding the increase in length ΔL for each joint to the length of the extended middle finger (see Table A1) gives the length of skin that spans the middle finger when it is flexed actively to the maximum (see Table A4).

In order to validate the chosen sensor concept, it is necessary to determine the distance between the MCP and PIP joints of the middle finger for different percentiles. In the course of reviewing the literature for this paper, no study was found that provides absolute values for the lengths of the individual phalanges. Instead, [49] gives ratios for distances between finger joints.

For the distance $\bar{M_{PIP}M_{MCP}}$ between the centers M of the PIP and MCP joints, and the distance $\bar{M_{DIP}M_{MCP}}$ between the centers M of the DIP and MCP joints, the relationship according to Equation (5) applies:

(5) $$\bar{M_{PIP}M_{MCP}}=\left(0.58\pm 0.035\right)\;\cdot \;\bar{M_{DIP}M_{MCP}}$$

The constant 0.58 is the average ratio between the distances. It has a standard deviation of 0.035. For an approximate calculation of the joint distances, the unknown distance $\bar{M_{DIP}M_{MCP}}$ is assumed to be the total length of the relaxed finger. This allows the lengths of phalanges to be estimated upwards.

Table A5 presents the calculated results according to Equation (5) for the distance between the PIP and MCP joint, with the middle finger extended. The distance between the PIP and MCP joint with the middle finger flexed was determined by adding the increase in length for both joints from Table A3. The results are shown in Table A6.

### 2.5. Measurement Parameters

Using the proposed model of the hand (see Figure 1), it is possible to define measurement parameters that the wearable can record and that can be used to describe the kinematics of the hand. They are listed in Table 1.

Apart from the CMC joint in finger I, flexion and extension angles of all joints considered in the simplified kinematic model may be determined.

In addition, SenGlove was equipped with additional sensors to measure further parameters for possible future applications. SenGlove can detect contact pressure at all fingertips. While the handling of objects is not intended with SenGlove at this point of the development, this information can be used to determine whether the fingertips of individual fingers touch the palm of the hand or the thumb (cp. Section 2.1). If contact pressure is detected for all four long fingers, this is evidence that the wearer’s hand has been closed. In the case of finger I, this means that the thumb is in opposition. While the basic assumption described in Section 2.2 and Equation (1) is only valid without external forces, with a different assumption, a more complex fusion of data or the combined evaluation of the DIP and PIP joint, the detection of object contact is also possible.

Angular velocity and linear acceleration are measured in all three axes, each at the back of the hand, to determine the movement of the whole hand. An absolute orientation/rotation vector describes the orientation of the hand in space (see Section 2.6).

### 2.6. Sensor Concept

**Figure 4:**
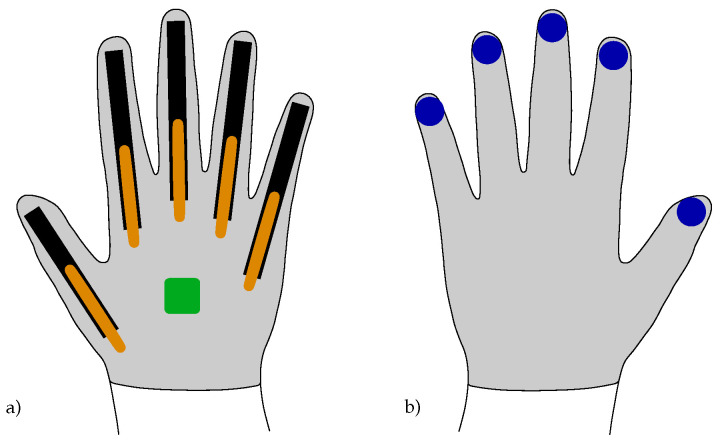
Final sensor concept of SenGlove. Variant shown: small-sized hands with a textile support structure for the hand (grey). (**a**) dorsal view with inertial measurement unit (green), bending sensors for the flexion of an entire finger (black), bending sensors for the flexion of the MCP joint (orange), (**b**) palmar view with pressure sensors (blue).

The sensor principle of SenGlove is based on a total of ten flex sensors, five pressure sensors, and one IMU (see Figure 4).

Flex sensors were chosen as a result of literature research to minimize the need for additional mechanical components. Furthermore, they promise the highest accuracy in combination with low weight and easy handling. We use the flex sensors to determine the flexion and extension of all finger joints individually. With the biomechanical linear constraint described in Section 2.2 and Equation (1), the DoF per finger and with that number of needed sensors is reduced from three to two. Yet both the flexion and extension of individual joints and the sum of several joints still have to be measured to determine the flexion and extension angles of each joint of a finger. Therefore, different lengths and different active lengths of the sensors are necessary. Different lengths are needed also for the adaption of the concept to different hand sizes (cp. Section 2.4).

**Figure A2:**
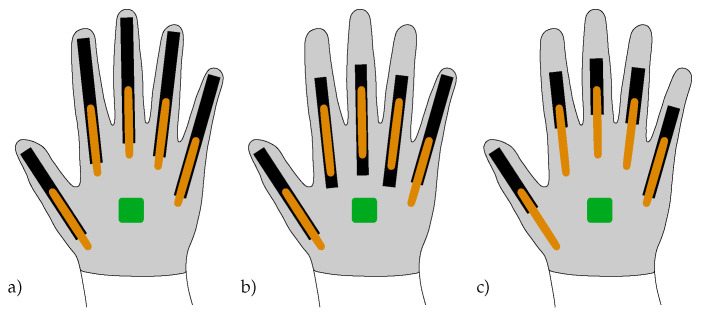
Position variants of the flex sensors (black), depending on the size of the user’s hand in dorsal view. Additional elements shown: mechanical support structure (grey), inertial measurement unit (green), bending sensors for the flexion of the *Metacarpophalangeal* joint (orange). (**a**) Variant for small-sized hands (**b**) Variant for medium-sized hands (**c**) Variant for large-sized hands.

There are five long and five short flex sensors, two sensors for each finger. The long sensors are realized with *1-Axis Soft Flex Sensors* from Bend Labs^®^, and the short sensors with flex sensors (*FSL-0055-253-ST*) from Spectra Symbol^®^. The Spectra Symbol^®^ sensors are placed over each MCP joint. They have an active length of 55 mm and therefore are long enough to cover the joint for every hand size (see Table A6). However, they are not too long to reach the PIP joint. Due to their limited permitted active length of 130 mm, three different position variants of the Bend Labs^®^ sensors are chosen, depending on the size of the user’s hand (see Figure A2). The variants consider the increase in length while bending the finger joints and the calculated distances between PIP and MCP joints (see Table A6).

We choose two different types of flex sensors because of the lengths required for the different measurement tasks. Furthermore, we assumed that the differing stiffnesses of the sensors help to minimize unwanted interactions between the sensors while moving the fingers.

The variant for small-sized hands allows the Bend Labs^®^ sensors to span the MCP, PIP and DIP joints of the fingers II to V and the MCP and IP joints of finger I (see Figure A2a). They measure the combined flexion and extension angles of three and two joints, respectively. This variant is even suitable for women of the first percentile.

In the variant for medium-sized hands, the Bend Labs^®^ sensors span the MCP and PIP joints of Finger II to V (see Figure A2b). This variant fits men up to the 99th percentile. From the 5th percentile for men and the 95th percentile for women, a stretching of the sensors is necessary to compensate for the increase in length.

The variant for large-sized hands is suitable, on the one hand, for wearers whose hands are larger than those of the 99th percentile of men. On the other hand, it offers an alternative for users of the variant for medium-sized hands, where the sensors stretch when the fingers are flexed. With this variant, the Bend Labs™ sensors span the PIP joint of the fingers II to V and the IP joint of finger I (see Figure A2c).

Pressure sensors are installed on the wearable’s fingertips to detect the touch of the fingers with the palm and the opposition position of the thumb (cp. Section 2.1 and Section 2.5). The force sensing resistors (FSR) are used as digital switches. Those switches are toggled when the electrical resistance of the sensors has reached a threshold value.

The IMU sensor is mounted flat on the back of the wearable’s hand to detect the orientation of the user’s hand in space. The Adafruit^®^ *9-DOF Orientation IMU Fusion Breakout—BNO085* used here has an "absolute" orientation/rotation vector as direct output. This vector is a four-point quaternion output for accurate data manipulation gained via fusion of the accelerometer, gyroscope (and magnetometer) data—see https://www.adafruit.com/product/4754 (accessed on 31 January 2023) for further details. While the coordinate system of the IMU is assumed to be congruent to the coordinate system of the hand, the absolute orientation vector can be projected onto the hand and describes its orientation in space.

### 2.7. Mechanical Structure

**Figure 5:**
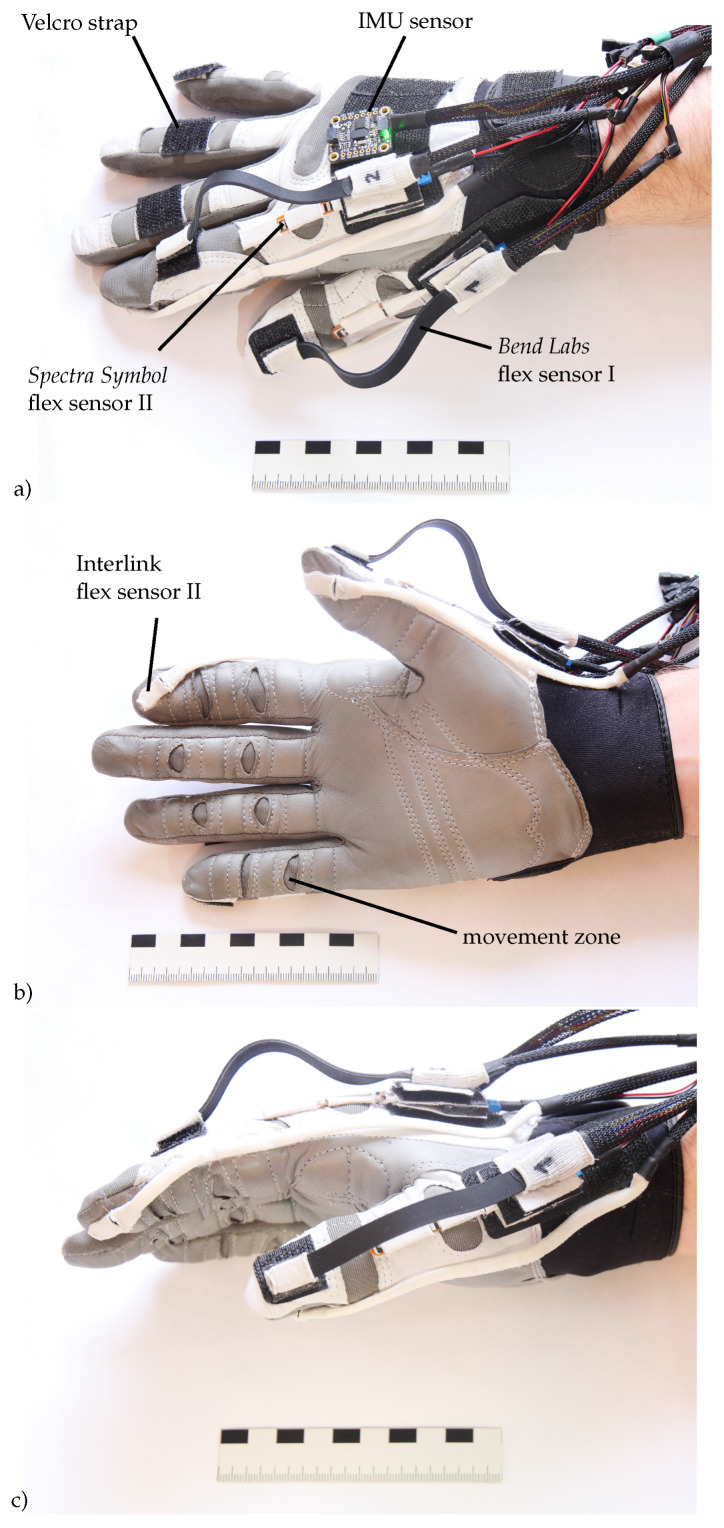
Final realization of SenGlove for a medium-sized right hand with two fingers equipped with sensors. (**a**) Dorsal view. (**b**) Palmar view. (**c**) Lateral view.

For SenGlove, a support structure consisting of a textile glove was selected (cp. design variants in Appendix A). The chosen glove is a ReliefGrip™ from Bionic Glove Technologies [50]. The glove chosen is equipped with movement zones made of thin breathable fabric; the zones are placed above each finger joint to minimize the restriction of freedom of movement of the fingers (see Figure 5b). Therefore, we assume Equation (1) to be valid. A Velcro strap prevents the glove from slipping off the hand (see Figure 5a). The glove is commercially available for both men and women in six different sizes. Each version can be used for the wearable.

Two different attachment methods for the sensors are used. On one side, the pressure sensors were put into tubes made of elastic fabric, which were glued directly onto the glove (see Figure 5b). On the other side, in addition to the elastic tubes, Velcro strips were used for the flex sensors and the IMU sensor (see Figure 5a). All electronic components can be detached from the glove so that it is machine washable.

**Figure 6:**
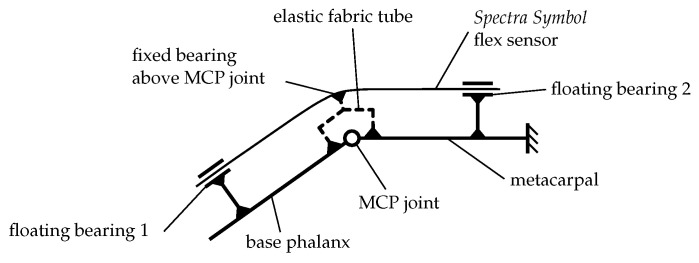
Principle of sensor mounting for the Spectra Symbol^®^ flex sensor. A mixture of fixed and floating bearings secures a symmetrical bending of the sensor over the joint. Shown for the *Metacarpophalangeal* (MCP) joint.

The electrical connectors of the two flex sensors on each finger are stacked on each other. Nevertheless, the mountings of the two sensor types differ. Fixed and floating bearings are used for the Spectra Symbol^®^ sensor (see Figure 6). The fixed bearing is located exactly above the MCP joint of a finger. It consists of a tube of elastic fabric glued to the glove. The diameter of the tube is selected in a way that the sensor is clamped in it and can not slide. The position of the fixed bearing guarantees that the axis of rotation of the flex sensor has a constant position above the MCP joint. The sensor is inserted into the fixed bearing in such a way that the bearing is in the center of the sensor. As a result, the bending of the sensor is symmetrical to its center and occurs over the entire active length. The floating bearings of the sensor mounting are located at the sensor ends. They consist of inelastic material, through which the sensor is pushed, and which has no clamping effect. The tube for the distal sensor end is directly glued onto the glove. The tube for the proximal sensor end is fixed with a Velcro strap so that its position is easily adjustable. Due to the two floating bearings at the ends of the sensor, the sensor can move relative to the glove when the fingers are bent, compensating for the increase in length ΔL during finger flexion.

No floating bearings are required for the Bend Labs^®^ sensors, as their flexibility allows them to stretch and compress. Fixed bearings made of elastic tubes are used at both sensor ends. The bearings are fixed with Velcro straps. The distal bearing is placed directly on the glove. The proximal bearing is on top of the electrical connector of the Spectra Symbol^®^ sensor. The sensors are placed in such a way that they do not stretch when the fingers are flexed to the maximum extent. Instead, the sensors are compressed and lift off the fingers in an arc when the fingers are stretched.

**Figure 7:**
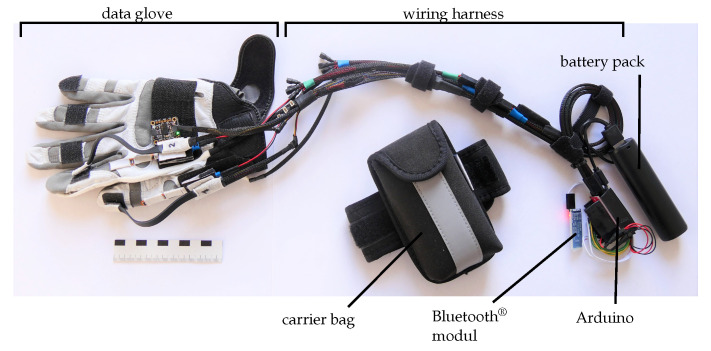
Overview of SenGlove. Realization shown for a medium-sized right hand with two fingers equipped with sensors. Carrier bag used to store the battery pack, and the Arduino can be strapped to the upper arm.

For optimized wearing comfort, the components of the wearable with the largest mass are worn proximally on the arm. The microcontroller and the battery pack are stowed in a carrying bag that is strapped around the upper arm (see Figure 7).

### 2.8. Electronic Structure

Further electronic components of the proposed wearable are the following:

- Microcontroller (Arduino Nano^®^ *RP2040 Connect*);
- Bluetooth^®^ module (*HC-06*);
- Pressure sensors (Interlink Electronics^®^ *FSR 400 Short*);
- IMU sensor (Adafruit^®^ *9-DOF Orientation IMU BNO085*);
- Low current lithium-ion battery pack (Tenergy^®^ *51126*).

**Figure 8:**
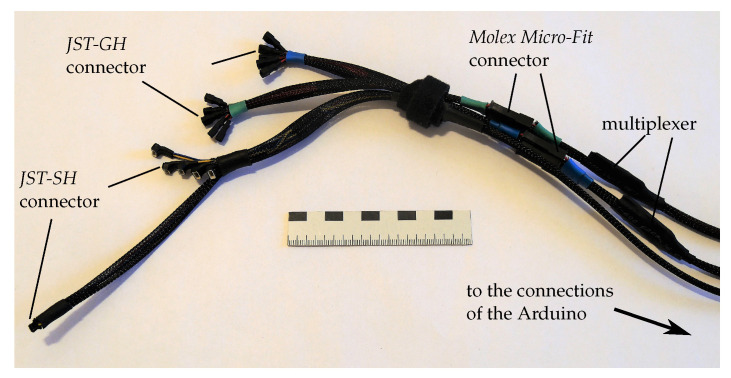
Wiring harness of SenGlove.

**Figure 9:**
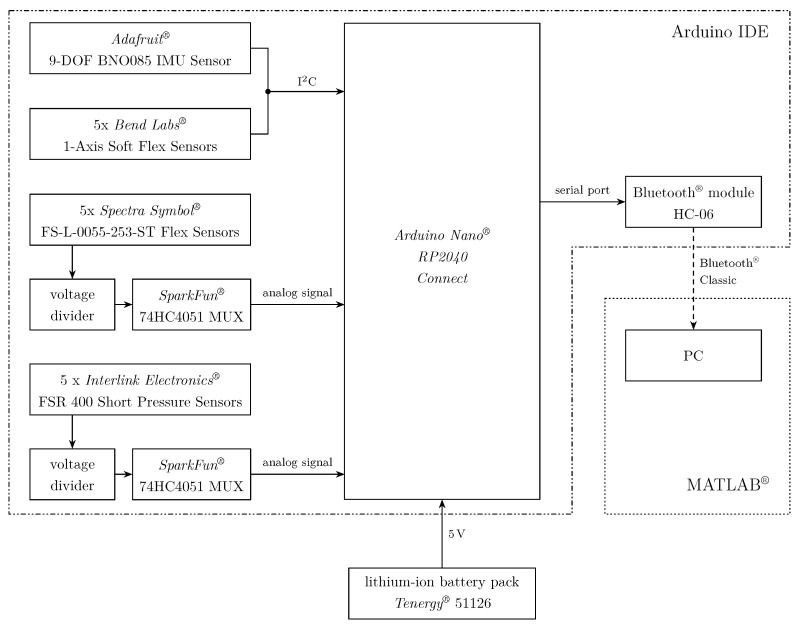
Electronic components and signal structure of SenGlove.

All sensors of SenGlove are connected to the Arduino via a three-part wiring harness (see Figure 8). The Arduino microcontroller is used to read out the sensors and to send their measurement data wirelessly to a personal computer (PC) using an additional Bluetooth^®^ module. All connections between the electric components are shown in Figure 9. The Bluetooth^®^ module uses the Bluetooth^®^ classic protocol. With this protocol, a higher transmission rate could be achieved compared to the Bluetooth^®^ Low Energy (BLE) protocol used by the Bluetooth^®^ module onboard of the Arduino Nano^®^ *RP2040 Connect*.

Voltage dividers and the internal analog-digital converter (ADC) of the Arduino are used to read out the analog signals of each Spectra Symbol^®^ sensor and pressure sensor, respectively. Since the Arduino has a total of eight analog inputs, the five flex and pressure sensors can not be connected directly to the Arduino. Therefore, two multiplexers are installed, which are used to read the signals successively via one analog input each.

The Bend Labs™ sensors have a direct interface to an $I^{2}C$ bus, and do not require further electronic components to be read out.

All electronic components of the wearable are powered by a lithium-ion battery pack, which is connected to the USB port of Arduino. The battery pack provides a maximum current of 1 A at a voltage of 5 V. The Bend Labs^®^ sensors, the IMU sensor and the Arduino work with an operating voltage of 3.3 V. The onboard step-down converter of the Arduino is used to convert 5 V to 3.3 V. The Spectra Symbol^®^ sensors, the pressure sensors and the corresponding voltage dividers are powered by 5 V.

### 2.9. Software

Two kinds of programs are implemented for the wearable. One is an Arduino sketch, which runs on the Arduino. The other one is a MATLAB^®^ script, which runs on a PC that is connected to the Bluetooth^®^ module.

The Arduino sketch is written with the Arduino IDE (version 1.8.15). The sketch is used to read the sensors of SenGlove, perform the necessary operations to calculate the angles of each finger joint, and send the data of all sensors to a PC via the Bluetooth^®^ module. The electronic components associated with each program are marked in Figure 9.

The MATLAB^®^ script is written with MATLAB^®^ version R2021a. The script creates a graphical user interface (GUI) through which the collection of the measurement data can be controlled. For each of the five fingers, for the accelerometer and the gyroscope of the IMU, there are plots provided in which the corresponding data are displayed. The GUI can also be used to save the data. Besides the plots, there is the option to animate the temporal progression of the angle changes by a 3D model of a hand (see Section 3.4). The MATLAB^®^ Toolbox *SynGrasp* is used to implement the animation [51].

### 2.10. Validation Method

To validate the measurement accuracy and runtime of SenGlove, several measurements were performed with a setup where fingers I to III were sensorized. During the measurements, the wearable was connected to a PC via cable and the USB interface of the Arduino.

#### 2.10.1. Measurement Accuracy

**Figure A3:**
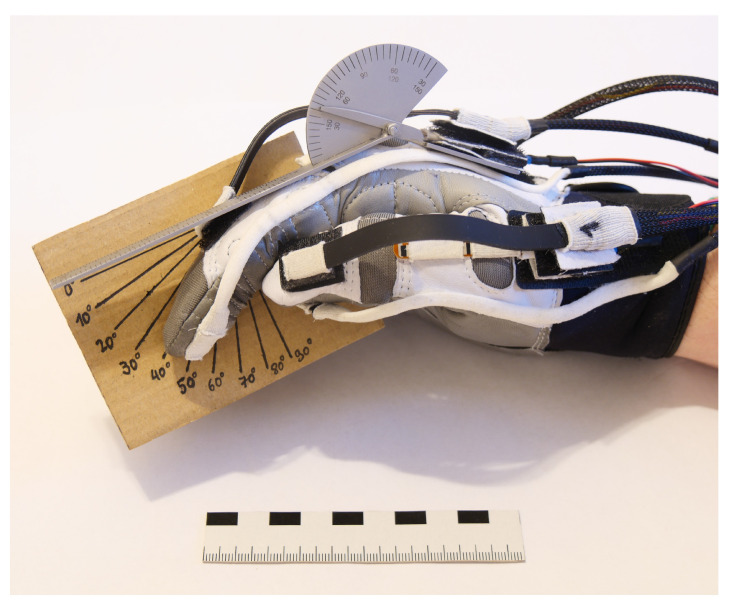
Validation setup for SenGlove. Using an analog finger goniometer, the target angles $\theta_{MCP,set}$ and $\theta_{PIP,set}$ were set at the *Metacarpophalangeal* (MCP) and *Proximal Interphalangeal* (PIP) joint of the finger and kept constant during a series of measurements. In this figure, a measurement for finger II with $\theta_{MCP,set}=40{}^{\circ}$, $\theta_{PIP,set}=30{}^{\circ}$ is shown. Compliance with these angles was checked using the finger goniometer and an angle template. After completion of the validation measurement, the angle of the *Distal Interphalangeal* (DIP) joint was measured with the finger goniometer, which was set due to the coupling to the PIP joint.

All angle measurements were performed according to the neutral-zero method [39]. For finger I, the MCP joint of the thumb is flexed at an angle $\theta_{MCP,set}$, which remains unchanged throughout the series of measurements. The value of the angle can assume multiples of ten in a measurement range from 0° to 50°. Subsequently, the IP joint is flexed in 10 steps in a range from 0° to 90°. At each step, the wearer keeps the position of his thumb constant while the wearable takes 50 measurements at 100 ms intervals. An analog finger goniometer was used to set the angles (see Figure A3).

For fingers II and III, the procedure was analogous to finger I. The measuring range for the MCP joint is between 0° and 70° and 0° and 80°, respectively. The angle was increased in 10° steps for each new measurement series. The angle of the PIP joint was increased from 0° to 90° in 10° increments. The angle was initially set with an analog finger goniometer and controlled by an angle template during the measurement (see Figure A3). To complete the series of measurements, the angle of the DIP joint was measured. Since the DIP and PIP joints are coupled with each other, no angle $\theta_{DIP,set}$ was specified for this joint. Instead, the angle of the DIP joint was assumed after $\theta_{PIP,set}$ was set, which was directly measured with an analog finger goniometer.

#### 2.10.2. Runtime

Runtime measurements for the Arduino sketch were performed to validate the software. The Arduino was connected with the receiver PC via Bluetooth^®^ Classic, and the sensor data sent were received by the MATLAB^®^ script. A transmission confirmation message was implemented in the Arduino sketch to measure the transmission delay between the Bluetooth^®^ module and the receiver PC. This message is displayed on the serial monitor when the transmission of a data packet via Bluetooth^®^ is completed. In addition, the MATLAB^®^ script sends a message to the Arduino confirming the reception of the packet as soon as a data packet has been read out from the serial port. This reception confirmation is also displayed on the serial monitor. The Arduino will not give out a new sent confirmation until it has received a confirmation of reception. The time difference between the two messages can be determined by the time stamps of the serial monitor. Transmission delay is half of this time difference, assuming that the transmission of the data packet takes the same time as the transmission of the confirmation.

## 3. Results

### 3.1. Final Design

**Figure A7:**
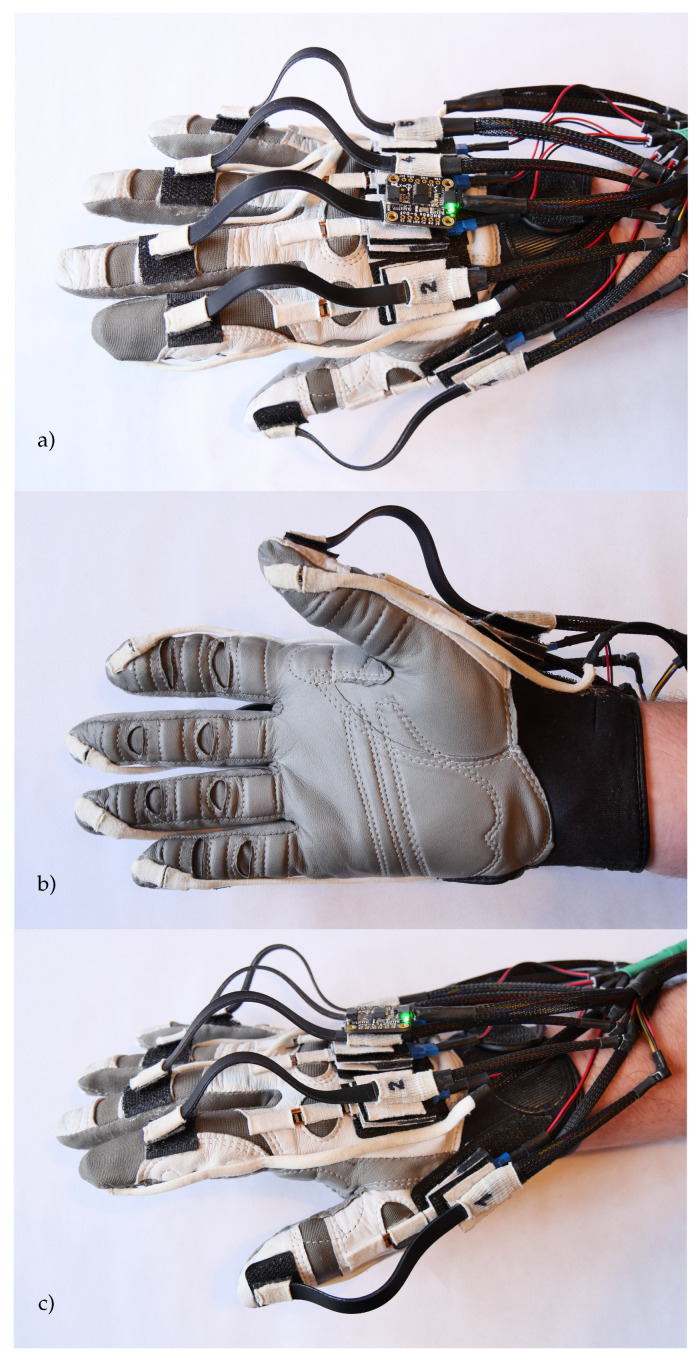
Final realization of SenGlove for a medium-sized right hand with all five fingers equipped with sensors. (**a**) Dorsal view. (**b**) Palmar view. (**c**) Lateral view.

The final design of SenGlove—with sensors for fingers I and II—is shown in Figure 7 and Figure 5 for a medium-sized right hand (cp. Section 2.6 and Figure A2). It sensorizes the MCP and IP joints of finger I and the MCP, PIP and DIP joints of finger II. It can measure the flexion and extension angles of each of these joints with a maximum root-mean-square error (RMSE) of less than 2.4° (see Section 3.3). In the full setup of SenGlove (see Figure A7), ten flex sensors, five pressure sensors, and an IMU with an accelerometer and a gyroscope are used. Fingers III to V are equipped and considered analogous to finger II.

All sensors are connected to the Arduino microcontroller. The sensor data are transmitted via Bluetooth^®^ Classic to a PC, which runs a MATLAB^®^ Script (see Section 2.9). It generates a GUI and a plot for each finger, which displays the data as a live stream. As an alternative to display the data, a 3D animation of the measured hand movement can be generated (see Section 3.4).

The system is modular so that only the sensors that are required for the specific task need to be installed. The sensors are attached to a glove that acts as a textile support structure. The size of the glove and the position of the sensors can be adjusted depending on the size of the wearer’s hand (see Section 2.4 and Section 2.6). The wearable can be fitted to a woman in the 5th percentile and a man in the 95th percentile.

### 3.2. Power Consumption and Mass

SenGlove is powered by a battery pack at 5 V. The maximum current used by SenGlove is 30 mA. Therefore, the power consumption of the wearable is 150 mW. The battery pack has a capacity of 2500 mAh. Thus, battery life of the wearable is theoretically approximately 83 h with a fully charged battery.

The total mass of the SenGlove with all its components is 270 g. Most of the mass is contributed by the components in the carrying bag on the wearer’s upper arm, which weighs 177 g, including the bag itself. The glove with sensors attached but without the wiring harness weighs 58 g.

### 3.3. Measurement Accuracy and Runtime

The single results for the achieved measurement accuracy are shown in Appendix E (Finger I), Appendix F (Finger II) and Appendix G (Finger III). The reference lines in the plots show the course of the data for an ideal measurement with no measurement deviation. Due to the stiffness of the ReliefGrip™ glove, not all angle combinations could be set while using the wearable. Therefore, the results do not cover the full range of values.

**Figure 10:**
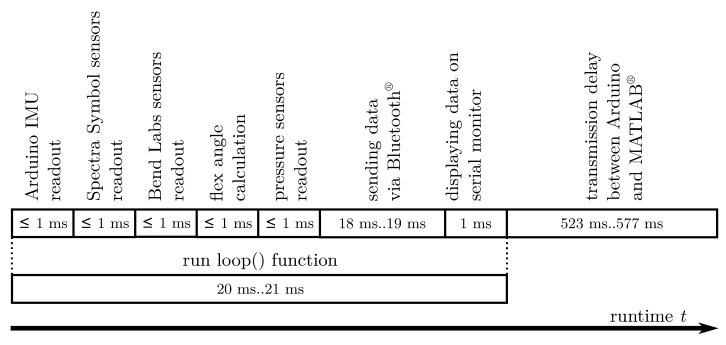
Timeline with runtime measurement results for the Arduino sketch.

Table 2 illustrates the combined RMSE for finger I to III, depending on the joint. Especially for finger I and the MCP and PIP joints of finger II and finger III, low RMSE values of less than 1° can be shown. Only the measurements of the DIP joints and also the combined value for the MCP, PIP and DIP joints show a higher RMSE with values up to 2.39°. Figure 10 shows a graphical illustration of the results of the runtime measurements (see Section 2.10) as a timeline for each sensor read out, sending and displaying the data. All measurement parameters can be read out and calculated in less than 21 ms per loop.

The longest time span of 523 to 577 ms appears as a transmission delay between Arduino and MATLAB^®^. The achieved run time of 20 ms to 21 ms per loop results in a sampling frequency of 47 Hz to 50 Hz for SenGlove in general.

### 3.4. Variants of Visualization

**Figure 11:**
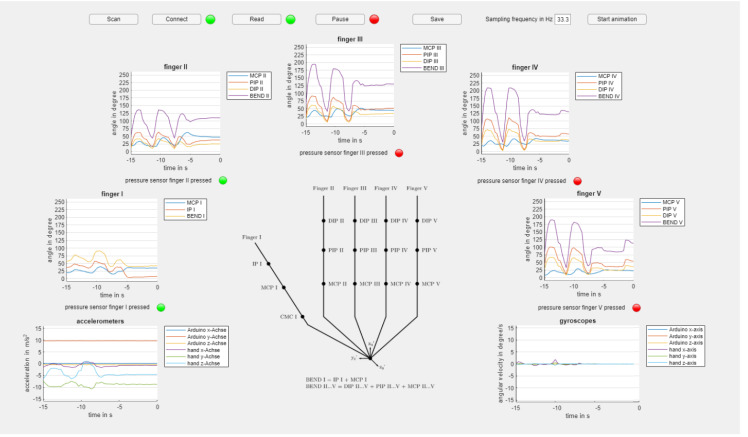
GUI of the MATLAB^®^ script for SenGlove. Menu at the top to scan for devices (Scan), connect to a device (Connect), start reading data (Read), pause reading data (Pause), store read data (Save), display of the current sampling frequency (Sampling frequency) and to start the 3D animation of the hand (Start animation). In center: Simplified kinematic model of a human hand with a degree of freedom of 15. Fingers numbered I (thumb) to V (pinkie finger). Joints: *Carpometacarpal* (CMC), *Distal Interphalangeal* (DIP), *Interphalangeal* (IP), *Metacarpophalangeal* (MCP), *Proximal Interphalangeal* (PIP). Global coordinate system with the axes $x_{0}^{{}^{\prime }}$, $y_{0}^{{}^{\prime }}$ and $z_{0}^{{}^{\prime }}$. Subplots (from center to right) for the linear acceleration of the hand (accelerometers), angle in degree for Finger I to V with pressure information for each finger underneath (green = threshold exceeded, red = threshold not exceeded), angular velocity of the hand (gyroscopes).

The implemented MATLAB^®^ script for SenGlove generates a GUI that offers two variants for visualizing the data. While running measurements, the GUI displays the data of the IMU sensor and the flex sensors in seven different live plots (see Figure 11). The status of the pressure sensor at each fingertip is displayed via a light. If a sensor is pressed, the corresponding light turns green; if not, the light is red. Transmission delay between the Arduino and the PC that is running the MATLAB^®^ script is less than 600 ms (see Figure 10). The data of the live plots can be stored for further investigation and visualization.

**Figure 12:**
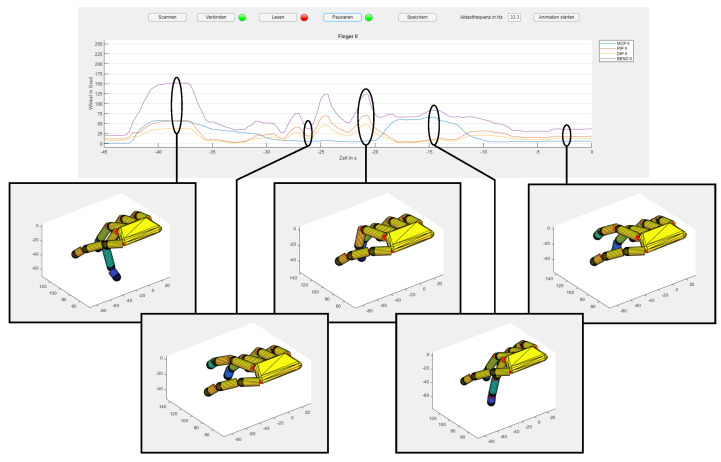
Series of screenshots of the 3D animation of a hand model using the MATLAB^®^ toolbox SynGrasp [51]. Finger II has been flexed and extended. Data stream shown in the subplot at the top with angle value (in degree) for the *Distal Interphalangeal* (DIP), *Metacarpophalangeal* (MCP), *Proximal Interphalangeal* (PIP) joint as well as overall bending of the finger (Bend II).

Based on the measured joint angles, a 3D animation of a moving hand can be generated. It displays the temporal progression of the angle changes in three-dimensional space (see Figure 12).

## 4. Discussion

With the presented wearable SenGlove, it is possible to measure 14 of 15 angular values of the simplified hand model (see Section 2.2). Additionally, five contact pressure readings as well as inertial data of the whole hand with a DoF of 6 can be measured. With SenGlove, it is possible to determine the angles of all finger joints of the hand digitally and simultaneously. We reduced the number of needed sensors for that task as well as the complexity of the necessary calculations in comparison to existing solutions—especially using IMUs (cp. [30,32]). The RMSE achieved for the joint angles on finger I is 0.99°, for finger II 1.53° degrees and for finger III 2.38°. The accuracy for fingers IV and V is assumed to be comparable to the results shown for fingers II and III. The results show that despite the simplifications made, a high measurement accuracy could be achieved with commercially available components. According to *Durlach et al. 1995*, the human perception threshold for the angles of the finger joints is 2.5° [42]. The results show that the wearable’s measurement accuracy for each joint is below this threshold. Accordingly, the wearable is able to measure the angles of the finger joints more accurately than a person’s own perception of them. In the literature, there are only a few implementations that can measure all finger joints of the hand. Their measurement accuracy varies greatly. Most results are between 2 and 7 [29,30,31,32,34]. *Hsio et al. 2015* achieved the highest angular accuracy with an average error of 0.98° [33]. However, this result was only achieved on a mechanical test rig and only for one pair of sensors. The sensor concept was not validated with the designed wearable on a human hand. Compared with the investigated sources, SenGlove demonstrates the highest measurement accuracy for measuring finger joint flexion angles on a non-visual basis in an application without a test rig.

The use of flex or bending sensors is already rated very good in the literature in comparison to other vision, encoder or IMU-based systems (cp. [30]). The main advantages are the low cost, high wearability, and portability. Thus far, bending sensors lack the accuracy and repeatability of other systems. With our contribution, we showed ways to minimize these disadvantages.

However, the measurement results obtained must be considered in relation to the validation method used. An analog finger goniometer was used, with a measuring scale divided into 5° sections. The basic assumption for the reading uncertainty of a measuring instrument corresponds to half of the smallest scale unit. In the case of the finger goniometer used, angle measurements can be performed with an uncertainty of 2.5° (without taking into account the compliance in coupling the device to the finger, which will not be discussed here since no gold standard exists). With the designed wearable, measured values can be specified with up to two decimal places. It is not possible with the analog finger goniometer to validate the measurement results of the wearable with absolute certainty.

Since the proposed wearable is intended to be used by many people and only minimal individual adaptation to the wearer should be required, the approximated linear relationship from Equation (1) is used. This constraint can only be seen as a simplified approximation and is only valid without external forces. Among others, *Park et al. 2015* investigated the relationship between the joints, and was able to show that it can be described more precisely with a second-degree polynomial [45]. Moreover, the coefficients of this polynomial are different for each person and should be determined individually through experiments.

We proposed a modular design. The textile support structure can be adapted to the hand size of the wearer as well as the position of the sensors. The wearable can be equipped with sensors for any combination of fingers of the hand. This helps save and adjust costs when only some fingers have to be measured. Figure A7 shows the proposed wearable with sensors for five fingers attached (cp. Figure 5). Furthermore, the results show that the glove used restricts the range of motion of the finger joints due to the lower deformability of the support structure compared to the human skin.

## 5. Conclusions and Outlook

In this contribution, we show that—although using only commercially available components—(bio-)mechatronic principles [40,41] can be utilized to create a cost-effective and size-adaptable design for a wearable for the human hand with a high measurement accuracy (see Section 4 for details). Despite this fact, SenGlove can only be seen as a technical prototype and functional model so far.

For future technical applications (cp. Section 1.1), additional developments and optimizations have to be made; for example, to document rehabilitation progress, the range of motion of SenGlove has to be increased by using a more elastic and thinner support structure, e.g., made of rubber or silicone. To use SenGlove to translate sign language, problems such as user acceptance, the number of different languages and the need for large libraries or training have to be addressed [52]. Thus, the use of SenGlove as an input device for different technical applications, such as the programming or control of robots, seems to be closest.

Furthermore, SenGlove can be used as a platform for general technical developments or scientific research, e.g., the integration of additional sensor modalities or research on sensor fusion algorithms [53], deep learning [20] or different methods to determine specific gestures out of the raw data stream [54]. It could also be considered to combine the support structure with the sensors and use e-textile technology for the wearable (cp. [55,56,57,58]) or to discuss the integration of actoric components [13,24]. Additional challenges for such wearables, such as washability or the standardization of their development (cp. [3]), also need to be addressed in the future.

## Figures and Tables

**Table 1:** Recordable measurement parameter of SenGlove. Fingers numbered I (thumb) to V (pinkie finger). Joints: *Distal Interphalangeal* (DIP), *Interphalangeal* (IP), *Metacarpophalangeal* (MCP), *Proximal Interphalangeal* (PIP).

Finger	Measurement Parameter
I..V	flexion-/extension angle MCP joint
	(condition touching the palm with fingertip)
I	flexion-/extension angle IP joint
	(condition opposition position of finger I)
II..V	flexion-/extension angle PIP joint
	flexion-/extension angle DIP joint
**Hand**	**Measurement Parameter**
back of hand	absolute orientation/rotation vector
	(angular velocity in three axes)
	(linear acceleration in three axes)

**Table 2:** Root-mean-square error of all measurements for finger I (thumb) to finger III (middle finger). Joints: *Carpometacarpal* (CMC), *Distal Interphalangeal* (DIP), *Interphalangeal* (IP), *Metacarpophalangeal* (MCP), *Proximal Interphalangeal* (PIP).

Root-Mean-Square Error				
	**MCP joint**		**IP joint**	**MCP a. IP joint**
**Finger I**	0,96		1,03	0,99
	**MCP joint**	**PIP joint**	**DIP joint**	**MCP, PIP a. DIP joint**
**Finger II**	0,72	0,90	2,39	1,53
**Finger III**	0,32	0,50	2,34	2,38

**Figure A1:**
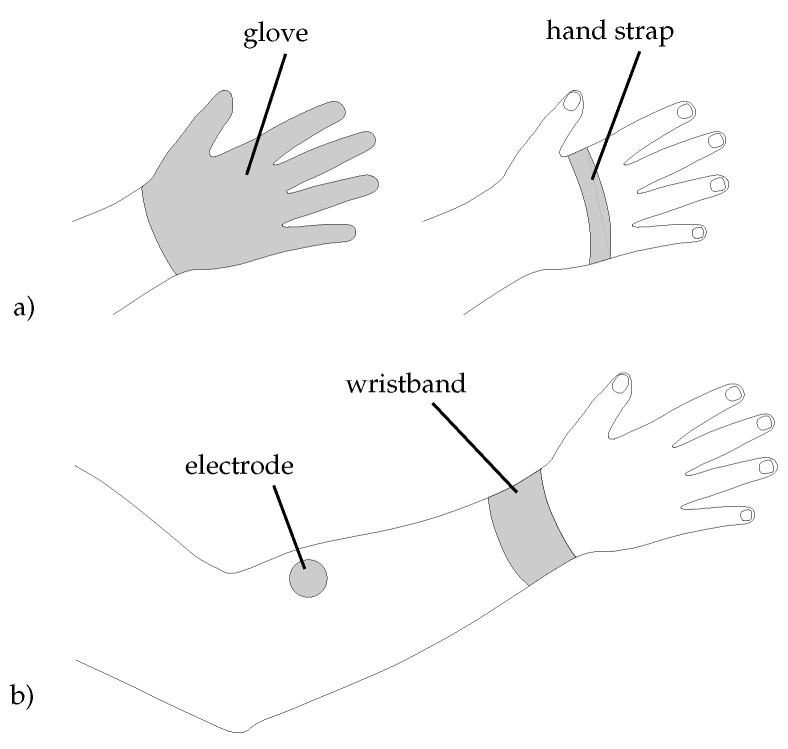
Types of wearables for the human hand or lower arm in dorsal view. Mechanical support structures shown in grey. (**a**) Wearables for the hand. Left: gloves. Right: hand straps. (**b**) Wearables for the lower arm using wrist straps or additional elements, such as electrodes for electromyography.

**Figure A4:**
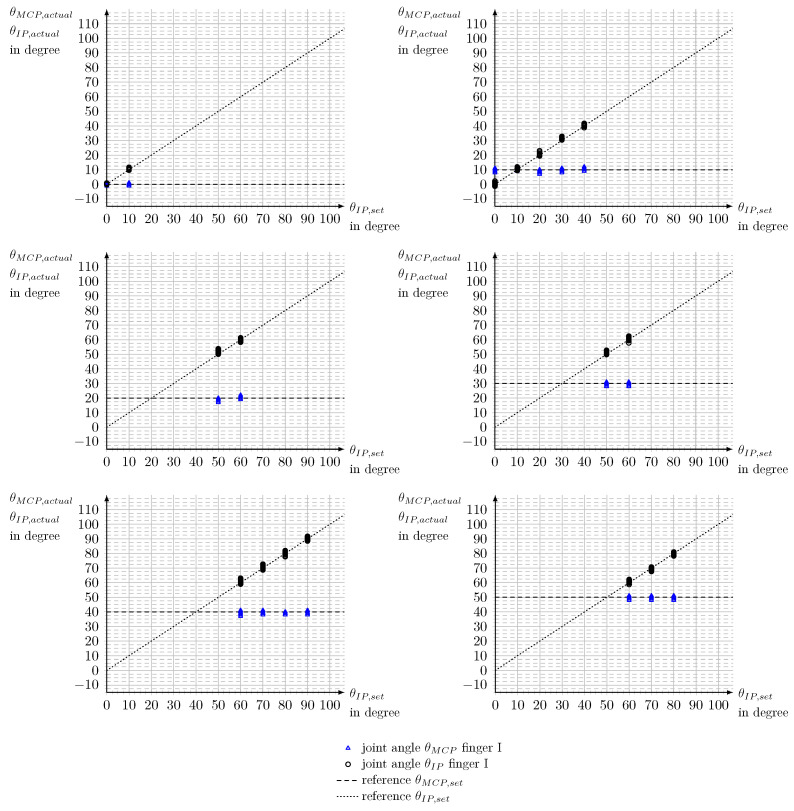
Accuracy of measurement for finger I. Joint angles θ set for the *Metacarpophalangeal* (MCP) joint from $\theta_{MCP,set}=0{}^{\circ}$ to $\theta_{MCP,set}=50{}^{\circ}$ and for the *Interphalangeal* (IP) joint $\theta_{IP,set}$. Joint angles $\theta_{MCP,actual}$ and $\theta_{IP,actual}$ measured with SenGlove.

**Figure A5:**
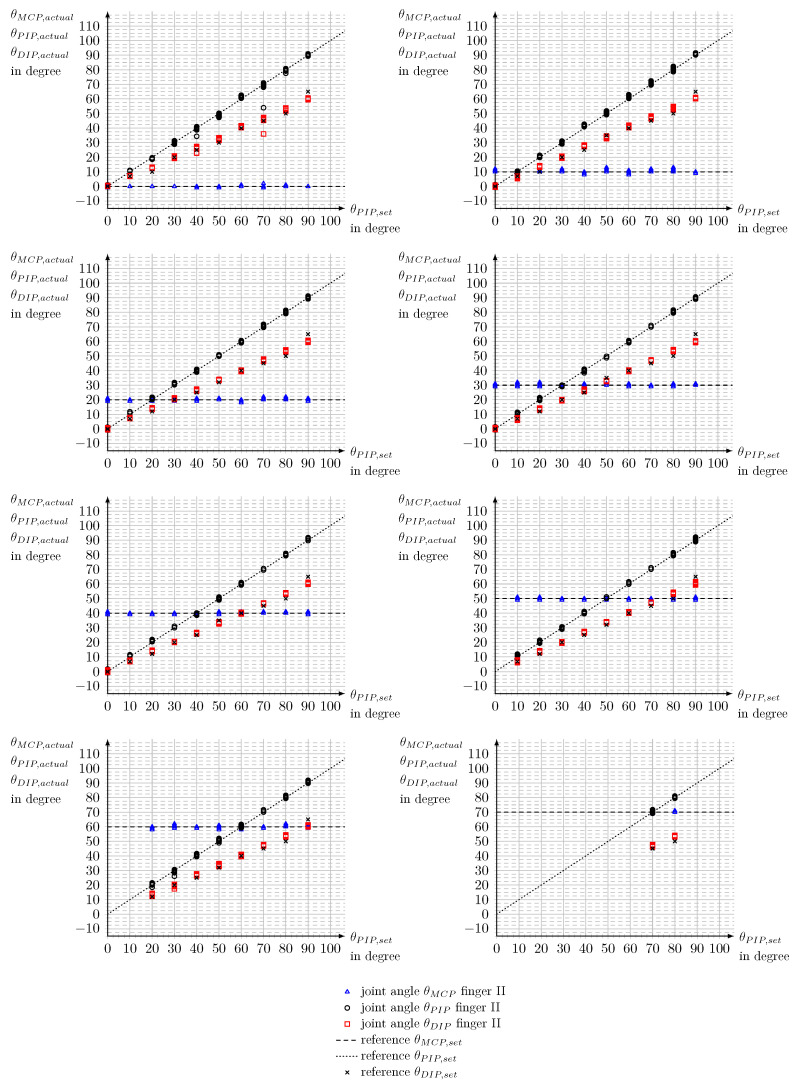
Accuracy of measurement for finger II. Joint angles θ set for the *Metacarpophalangeal* (MCP) joint from $\theta_{MCP,set}=0{}^{\circ}$ to $\theta_{MCP,set}=70{}^{\circ}$ and for the *Proximal Interphalangeal* (PIP) joint $\theta_{PIP,set}$. Reference for the *Distal Interphalangeal* (DIP) joint $\theta_{DIP,set}$ calculated in dependence of $\theta_{PIP,set}$. Joint angles $\theta_{MCP,actual}$ and $\theta_{PIP,actual}$ measured with SenGlove. $\theta_{DIP,actual}$ calculated in dependence of $\theta_{PIP,actual}$.

**Figure A6:**
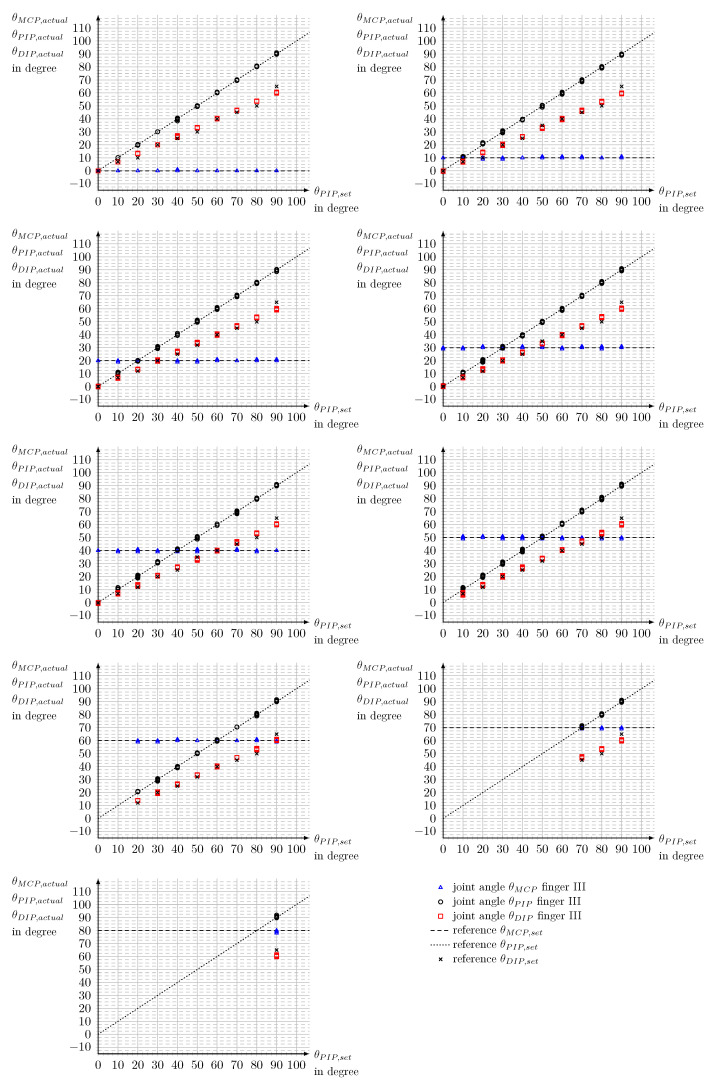
Accuracy of measurement for finger III. Joint angles θ set for the *Metacarpophalangeal* (MCP) joint from $\theta_{MCP,set}=0{}^{\circ}$ to $\theta_{MCP,set}=80{}^{\circ}$ and for the *Proximal Interphalangeal* (PIP) joint $\theta_{PIP,set}$. Reference for the *Distal Interphalangeal* (DIP) joint $\theta_{DIP,set}$ calculated in dependence on $\theta_{PIP,set}$. Joint angles $\theta_{MCP,actual}$ and $\theta_{PIP,actual}$ measured with SenGlove. $\theta_{DIP,actual}$ calculated in dependence on $\theta_{PIP,actual}$.

## Data Availability

The data presented in this study are available on request from the corresponding author.

## Abbreviations

The following abbreviations are used in this manuscript:

ADC	Analog–Digital Converter
BLE	Bluetooth^®^ Low Energy
CMC	Carpometacarpal
DH	Denavit–Hartenberg
DIP	Distal Interphalangeal
DOAJ	Directory of Open Access Journals
DoF	Degrees of Freedom
FSR	Force Sensing Resistor
GUI	Graphical User Interface
IDE	Integrated Development Environment
IMU	Inertial Measurement Unit
IP	Interphalangeal
MCP	Metacarpophalangeal
MDPI	Multidisciplinary Digital Publishing Institute
MUX	Multiplexer
PC	Personal Computer
PIP	Proximal Interphalangeal
USB	Universal Serial Bus
VDI	Verein Deutscher Ingenieure

## Appendix B. Finger Anthropometry

**Table A1:** Finger length of the extended middle finger for men and women, according to [46].

Finger Length of the Extended Middle Finger					
	**1st Percentile**	**5th Percentile**	**50th Percentile**	**95th Percentile**	**99th Percentile**
**Men**	102.0 mm	105.0 mm	114.0 mm	123.0 mm	127.0 mm
**Women**	89.0 mm	92.0 mm	101.0 mm	110.0 mm	114.0 mm

**Table A2:** Range of motion of the *Distal Interphalangeal* (DIP), *Metacarpophalangeal* (MCP), *Proximal Interphalangeal* (PIP) joints according to [39].

Range of Motion of the Finger Joints	
**Flexion/Extension**	**Finger II to V**
MCP	90°/0°/45°
PIP	110°/0°/0°
DIP	80°/0°/5°

**Table A3:** Radii of the middle finger joints *Distal Interphalangeal* (DIP), *Metacarpophalangeal* (MCP) and *Proximal Interphalangeal* (PIP), according to [46] and resulting increase in skin length over them.

Radii of the Middle Finger Joints and Resulting Increase in Length					
**Men**					
**Joint radius**	**1st percentile**	**5th percentile**	**50th percentile**	**95th percentile**	**99th percentile**
**MCP**	14.0 mm	14.7 mm	16.5 mm	18.3 mm	19.0 mm
**PIP**	8.5 mm	8.9 mm	10.0 mm	11.1 mm	11.5 mm
**DIP**	6.5 mm	6.9 mm	8.0 mm	9.1 mm	9.5 mm
**Increase in length**	**1st percentile**	**5th percentile**	**50th percentile**	**95th percentile**	**99th percentile**
**MCP**	22.0 mm	23.1 mm	25.9 mm	28.7 mm	29.8 mm
**PIP**	16.3 mm	17.1 mm	19.2 mm	21.3 mm	22.1 mm
**DIP**	9.1 mm	9.6 mm	11.2 mm	12.7 mm	13.3 mm
**Women**					
**Joint radius**	**1st percentile**	**5th percentile**	**50th percentile**	**95th percentile**	**99th percentile**
**MCP**	12.0 mm	12.6 mm	14.0 mm	15.4 mm	16.0 mm
**PIP**	7.5 mm	7.8 mm	8.5 mm	9.2 mm	9.5 mm
**DIP**	5.5 mm	5.8 mm	6.5 mm	7.2 mm	8.0 mm
**Increase in length**	**1st percentile**	**5th percentile**	**50th percentile**	**95th percentile**	**99th percentile**
**MCP**	18.8 mm	19.8 mm	22.0 mm	24.2 mm	25.1 mm
**PIP**	14.4 mm	15.0 mm	16.3 mm	17.7 mm	18.2 mm
**DIP**	7.7 mm	8.1 mm	9.1 mm	10.1 mm	11.2 mm

**Table A4:** Total length of skin over the middle finger at maximum flexion.

Total Length of Skin (Middle Finger, Maximum Flexion)					
	**1st Percentile**	**5th Percentile**	**50th Percentile**	**95th Percentile**	**99th Percentile**
**Men**	149.4 mm	154.8 mm	170.3 mm	185.5 mm	192.2 mm
**Women**	129.9 mm	134.9 mm	148.4 mm	161.9 mm	168.5 mm

**Table A5:** Distance between the *Proximal Interphalangeal* (PIP) and *Distal Interphalangeal* (DIP) joint with an extended middle finger.

Distance between the PIP and DIP joint (Extended Middle Finger)					
	**1st Percentile**	**5th Percentile**	**50th Percentile**	**95th Percentile**	**99th Percentile**
**Men**	59.2 ± 3.6 mm	60.9 ± 3.7 mm	66.1 ± 4.0 mm	71.3 ± 4.3 mm	73.7 ± 4.5 mm
**Women**	51.6 ± 3.1 mm	53.4 ± 3.2 mm	58.6 ± 3.5 mm	63.8 ± 3.9 mm	66.1 ± 4.0 mm

**Table A6:** Distance between the *Proximal Interphalangeal* (PIP) and *Distal Interphalangeal* (DIP) joint with flexed middle finger.

Distance between the PIP and DIP Joint (Flexed Middle Finger)					
	**1st Percentile**	**5th Percentile**	**50th Percentile**	**95th Percentile**	**99th Percentile**
**Men**	97.5 ± 3.6 mm	101.1 ± 3.7 mm	111.2 ± 4.0 mm	121.3 ± 4.3 mm	125.6 ± 4.5 mm
**Women**	84.8 ± 3.1 mm	88.2 ± 3.2 mm	96.9 ± 3.5 mm	105.7 ± 3.9 mm	109.4 ± 4.0 mm

## Appendix C. Denavit–Hartenberg Parameters

**Table A7:** Denavit–Hartenberg parameters for fingers I to V according to the newly introduced simplified hand model. Joint angle $\theta_{ji}$, joint distance $d_{ji}$, link length $a_{ji}$ and link twist angle $\alpha_{ji}$ for joint axis ji with finger number *j* and joint number *i*.

Denavit -Hartenberg Parameters				
**Joint *ji***	** $\theta_{ji}$ **	** $d_{ji}$ **	** $a_{ji}$ **	** $\alpha_{ji}$ **
j1	$\theta_{j1}$	0	$l_{j1}$	0°
j2	$\theta_{j2}$	0	$l_{j2}$	0°
j3	$\theta_{j3}$	0	$l_{j3}$	0°

## Appendix E. Measurement Accuracy of Finger I

**Table A8:** Results of the measurement series on the measurement accuracy of the joint angles of finger I. Measurement series sorted by dependence on the predetermined angle $\theta_{MCP,set}$ for the *Metacarpophalangeal* joint. Results shown are the maximum positive $\Delta \theta_{max}^{+}$ and negative $\Delta \theta_{max}^{-}$ deviations, as well as the root-mean-square error (RMSE). Joints: *Interphalangeal* (IP), *Metacarpophalangeal* (MCP).

Measurement Accuracy at Finger I			
**Measurement series $\theta_{MCP,soll}=0{}^{\circ}$**			
**Parameter**	**MCP joint**	**IP joint**	**MCP a. IP joint**
$\Delta \theta_{max}^{+}$	1°	1.69°	1.69°
$\Delta \theta_{max}^{-}$	0°	−0.31°	−0.31°
**RMSE**	0.56°	0.71°	0.64°
**Measurement series $\theta_{MCP,set}=10{}^{\circ}$**			
**Parameter**	**MCP joint**	**IP joint**	**MCP a. IP joint**
$\Delta \theta_{max}^{+}$	2°	3.01°	3.01°
$\Delta \theta_{max}^{-}$	−3°	−1.27°	−3°
**RMSE**	0.66°	1.00°	0.84°
**Measurement series $\theta_{MCP,set}=20{}^{\circ}$**			
**Parameter**	**MCP joint**	**IP joint**	**MCP a. IP joint**
$\Delta \theta_{max}^{+}$	2°	3.79°	3.79°
$\Delta \theta_{max}^{-}$	−3°	−1.71°	−3°
**RMSE**	1.08°	1.21°	1.15°
**Measurement series $\theta_{MCP,set}=30{}^{\circ}$**			
**Parameter**	**MCP joint**	**IP joint**	**MCP a. IP joint**
$\Delta \theta_{max}^{+}$	1°	2.76°	2.76°
$\Delta \theta_{max}^{-}$	−2°	−2.18°	−2.18°
**RMSE**	0.97°	1.22°	1.10°
**Measurement series $\theta_{MCP,set}=40{}^{\circ}$**			
**Parameter**	**MCP joint**	**IP joint**	**MCP a. IP joint**
$\Delta \theta_{max}^{+}$	1°	3.03°	3.03°
$\Delta \theta_{max}^{-}$	−3°	−2.20°	−2.20°
**RMSE**	1.19°	1.10°	1.14°
**Measurement series** $\theta_{MCP,set}=50{}^{\circ}$			
**Parameter**	**MCP joint**	**IP joint**	**MCP a. IP joint**
$\Delta \theta_{max}^{+}$	1°	2.23°	2.23°
$\Delta \theta_{max}^{-}$	−2°	−2.34°	−2.34°
**RMSE**	1.13°	0.87°	1.01°
**Measured values of all measurement series**			
**Parameter**	**MCP joint**	**IP joint**	**MCP a. IP joint**
$\Delta \theta_{max}^{+}$	2°	3.79°	3.79°
$\Delta \theta_{max}^{-}$	−3°	−2.34°	−3°
**RMSE**	0.96°	1.03°	0.99°

## Appendix F. Measurement Accuracy of Finger II

**Table A9:** Results of the measurement series on the measurement accuracy of the joint angles of finger II. Measurement series sorted in dependence on the predetermined angle $\theta_{MCP,set}$ for the *Metacarpophalangeal* joint. Results shown are the maximum positive $\Delta \theta_{max}^{+}$ and negative $\Delta \theta_{max}^{-}$ deviations, as well as the root-mean-square error (RMSE). Joints: *Distal Interphalangeal* (DIP), *Metacarpophalangeal* (MCP), *Proximal Interphalangeal* (PIP).

Measurement Accuracy at Finger II				
**Measurement series $\theta_{MCP,set}=0{}^{\circ}$**				
**Parameter**	**MCP joint**	**PIP joint**	**DIP joint**	**MCP, PIP a. DIP joint**
$\Delta \theta_{max}^{+}$	2°	2.55°	3.85°	3.85°
$\Delta \theta_{max}^{-}$	−1°	−16.06°	−5.42°	−16.06°
**RMSE**	0.46°	1.15°	2.43°	1.57°
**Measurement series $\theta_{MCP,set}=10{}^{\circ}$**				
**Parameter**	**MCP joint**	**PIP joint**	**DIP joint**	**MCP, PIP a. DIP joint**
$\Delta \theta_{max}^{+}$	3°	2.90°	4.87°	4.87°
$\Delta \theta_{max}^{-}$	−2°	−1.91°	−4.87°	−4.87°
**RMSE**	1.00°	1.18°	2.60°	1.75°
**Measurement series $\theta_{MCP,set}=20{}^{\circ}$**				
**Parameter**	**MCP joint**	**PIP joint**	**DIP joint**	**MCP, PIP a. DIP joint**
$\Delta \theta_{max}^{+}$	2°	1.76°	4.23°	4.23°
$\Delta \theta_{max}^{-}$	−2°	−1.05°	−5.57°	−5.57°
**RMSE**	0.71°	0.81°	2.25°	1.44°
**Measurement series $\theta_{MCP,set}=30{}^{\circ}$**				
**Parameter**	**MCP joint**	**PIP joint**	**DIP joint**	**MCP, PIP a. DIP joint**
$\Delta \theta_{max}^{+}$	2°	1.54°	4.36°	4.36°
$\Delta \theta_{max}^{-}$	−1°	−1.52°	−5.67°	−5.67°
**RMSE**	0.64°	0.62°	2.30°	1.43°
**Measurement series $\theta_{MCP,set}=40{}^{\circ}$**				
**Parameter**	**MCP joint**	**PIP joint**	**DIP joint**	**MCP, PIP a. DIP joint**
$\Delta \theta_{max}^{+}$	1°	1.85°	3.97°	3.97°
$\Delta \theta_{max}^{-}$	−1°	−1.42°	−5.15°	−5.15°
**RMSE**	0.65°	0.81°	2.24°	1.42°
**Measurement series $\theta_{MCP,set}=50{}^{\circ}$**				
**Parameter**	**MCP joint**	**PIP joint**	**DIP joint**	**MCP, PIP a. DIP joint**
$\Delta \theta_{max}^{+}$	1°	2.15°	4.30°	4.30°
$\Delta \theta_{max}^{-}$	−1°	−1.00°	−5.67°	−5.67°
**RMSE**	0.57°	0.71°	2.36°	1.46°
**Measurement series $\theta_{MCP,soll}=60{}^{\circ}$**				
**Parameter**	**MCP joint**	**PIP joint**	**DIP joint**	**MCP, PIP a. DIP joint**
$\Delta \theta_{max}^{+}$	2°	1.99°	4.36°	4.36°
$\Delta \theta_{max}^{-}$	−2°	−3.93°	−5.11°	−5.11°
**RMSE**	0.89°	0.92°	2.49°	1.62°
**Measurement series $\theta_{MCP,soll}=70{}^{\circ}$**				
**Parameter**	**MCP joint**	**PIP joint**	**DIP joint**	**MCP, PIP a. DIP joint**
$\Delta \theta_{max}^{+}$	1°	1.71°	4.05°	4.05°
$\Delta \theta_{max}^{-}$	−1°	−0,77°	1,16°	−1°
**RMSE**	0.61°	0.55°	2.79°	1.68°
**Measured values of all measurement series**				
**Parameter**	**MCP joint**	**PIP joint**	**DIP joint**	**MCP, PIP a. DIP joint**
$\Delta \theta_{max}^{+}$	3°	2.90°	4.87°	4.87°
$\Delta \theta_{max}^{-}$	−2°	−16,06°	−5.67°	−16.06°
**RMSE**	0.72°	0.90°	2.39°	1.53°

## Appendix G. Measurement Accuracy of Finger III

**Table A10:** Results of the measurement series on the measurement accuracy of the joint angles of finger III. Measurement series sorted in dependence on the predetermined angle $\theta_{MCP,set}$ for the *Metacarpophalangeal* joint. Results shown are the maximum positive $\Delta \theta_{max}^{+}$ and negative $\Delta \theta_{max}^{-}$ deviations, as well as the root-mean-square error (RMSE). Joints: *Distal Interphalangeal* (DIP), *Metacarpophalangeal* (MCP), *Proximal Interphalangeal* (PIP).

Measurement accuracy of finger III				
**Measurement series $\theta_{MCP,set}=0{}^{\circ}$**				
**Parameter**	**MCP joint**	**PIP joint**	**DIP joint**	**MCP, PIP a. DIP joint**
$\Delta \theta_{max}^{+}$	1°	0.92°	3.84°	3.84°
$\Delta \theta_{max}^{-}$	0°	−1.51°	−5.13°	−5.13°
**RMSE**	0.04°	1.30°	2.51°	1.46°
**Measurement series $\theta_{MCP,set}=10{}^{\circ}$**				
**Parameter**	**MCP joint**	**PIP joint**	**DIP joint**	**MCP, PIP a. DIP joint**
$\Delta \theta_{max}^{+}$	1°	1.65°	4.44°	4.44°
$\Delta \theta_{max}^{-}$	−1°	−1.40°	−5.68°	−5.68°
**RMSE**	0,20°	0.47°	2.44°	1.44°
**Measurement series $\theta_{MCP,set}=20{}^{\circ}$**				
**Parameter**	**MCP joint**	**PIP joint**	**DIP joint**	**MCP, PIP a. DIP joint**
$\Delta \theta_{max}^{+}$	1°	1.23°	3.63°	3.63°
$\Delta \theta_{max}^{-}$	−1°	−1.38°	−5.92°	−5.92°
**RMSE**	0.20°	0.39°	2.18°	1.28°
**Measurement series $\theta_{MCP,set}=30{}^{\circ}$**				
**Parameter**	**MCP joint**	**PIP joint**	**DIP joint**	**MCP, PIP a. DIP joint**
$\Delta \theta_{max}^{+}$	1°	1.20°	4.02°	4.02°
$\Delta \theta_{max}^{-}$	−1°	−1.28°	−5.52°	−5.52°
**RMSE**	0.35°	0.44°	2.12°	1.27°
**Measurement series $\theta_{MCP,set}=40{}^{\circ}$**				
**Parameter**	**MCP joint**	**PIP joint**	**DIP joint**	**MCP, PIP a. DIP joint**
$\Delta \theta_{max}^{+}$	1°	2.64°	3.63°	3.63°
$\Delta \theta_{max}^{-}$	−1°	−1.62°	−5.11°	−5.11°
**RMSE**	0.38°	0.62°	2.12°	1.29°
**Measurement series $\theta_{MCP,set}=50{}^{\circ}$**				
**Parameter**	**MCP joint**	**PIP joint**	**DIP joint**	**MCP, PIP a. DIP joint**
$\Delta \theta_{max}^{+}$	1°	2.26°	4.05°	4.05°
$\Delta \theta_{max}^{-}$	−1°	−1.31°	−5.27°	−5.27°
**RMSE**	0.50°	0.68°	2.19°	1.36°
**Measurement series $\theta_{MCP,soll}=60{}^{\circ}$**				
**Parameter**	**MCP joint**	**PIP joint**	**DIP joint**	**MCP, PIP a. DIP joint**
$\Delta \theta_{max}^{+}$	1°	1.55°	4.05°	4.05°
$\Delta \theta_{max}^{-}$	−1°	−1.21°	−4.99°	−4.99°
**RMSE**	0.26°	0.48°	2.18°	1.30°
**Measurement series $\theta_{MCP,soll}=70{}^{\circ}$**				
**Parameter**	**MCP joint**	**PIP joint**	**DIP joint**	**MCP, PIP a. DIP joint**
$\Delta \theta_{max}^{+}$	0°	1.74°	3.85°	3.85°
$\Delta \theta_{max}^{-}$	−1°	−0.50°	−5.28°	−5.28°
**RMSE**	0.33°	0.57°	2.69°	1.60°
**Measurement series $\theta_{MCP,soll}=80{}^{\circ}$**				
**Parameter**	**MCP joint**	**PIP joint**	**DIP joint**	**MCP, PIP a. DIP joint**
$\Delta \theta_{max}^{+}$	0°	1,85°	−3.76°	1.85°
$\Delta \theta_{max}^{-}$	−2°	−0.15°	−5.10°	−5.10°
**RMSE**	0.47°	0.46°	4.87°	2.83°
**Measured values of all measurement series**				
**Parameter**	**MCP joint**	**PIP joint**	**DIP joint**	**MCP, PIP a. DIP joint**
$\Delta \theta_{max}^{+}$	1°	2.64°	4.44°	4.44°
$\Delta \theta_{max}^{-}$	−2°	−1.62°	−5.92°	−5.92°
**RMSE**	0.32°	0.50°	2.34°	2.38°

## References

[1] Hirt B., Seyhan H., Wagner M., Zumhasch R. (2014). Anatomie und Biomechanik der Hand.

[2] Wood B., Collard M. (2001). The Meaning of Homo. Ludus Vitalis.

[3] Zaman S.u., Tao X., Cochrane C., Koncar V. (2022). Smart E-Textile Systems: A Review for Healthcare Applications. Electronics.

[4] Chakarov D., Veneva I., Tsveov M. A New Upper Limb Exoskeleton for Human Interaction with Virtual Enviroments and Rehabilitation Tasks. Proceedings of the 10th International Conference Mechatronic Systems and Materials (MSM 2014).

[5] Tawk C., in het Panhuis M., Spinks G.M., Alici G. (2019). Soft Pneumatic Sensing Chambers for Generic and Interactive Human–Machine Interfaces. Adv. Intell. Syst..

[6] Sun Z., Zhu M., Lee C. (2021). Progress in the Triboelectric Human–Machine Interfaces (HMIs)-Moving from Smart Gloves to AI/Haptic Enabled HMI in the 5G/IoT Era. Nanoenergy Adv..

[7] Abad A.C., Reid D., Ranasinghe A. (2022). A Novel Untethered Hand Wearable with Fine-Grained Cutaneous Haptic Feedback. Sensors.

[8] Balasubramanian S., Klein J., Burdet E. (2010). Robot-assisted rehabilitation of hand function. Curr. Opin. Neurol..

[9] Delph M.A., Fischer S.A., Gauthier P.W., Luna C.H.M., Clancy E.A., Fischer G.S. A soft robotic exomusculature glove with integrated sEMG sensing for hand rehabilitation. Proceedings of the 2013 IEEE 13th International Conference on Rehabilitation Robotics (ICORR).

[10] Lambercy O., Ranzani R., Gassert R., Colombo R., Sanguineti V. (2018). Chapter 15—Robot-assisted rehabilitation of hand function. Rehabilitation Robotics.

[11] Semprini M., Cuppone A., Squeri V., Konczak J. Muscle innervation patterns for human wrist control: Useful biofeedback signals for robotic rehabilitation?. Proceedings of the 2015 IEEE International Conference on Rehabilitation Robotics (ICORR).

[12] Duanmu D., Wang X., Li X., Wang Z., Hu Y. (2022). Design of Guided Bending Bellows Actuators for Soft Hand Function Rehabilitation Gloves. Actuators.

[13] Xia K., Chen X., Chang X., Liu C., Guo L., Xu X., Lv F., Wang Y., Sun H., Zhou J. (2022). Hand Exoskeleton Design and Human-Machine Interaction Strategies for Rehabilitation. Bioengineering.

[14] Zhou Z., Chen K., Li X., Zhang S., Wu Y., Zhou Y., Meng K., Sun C., He Q., Fan W. (2020). Sign-to-speech translation using machine-learning-assisted stretchable sensor arrays. Nat. Electron..

[15] Allela R., Muthoni C., Karibe D. (2019). Sign-IO. http://www.sign-io.com.

[16] Chen X., Gong L., Wei L., Yeh S.C., Da Xu L., Zheng L., Zou Z. (2021). A Wearable Hand Rehabilitation System With Soft Gloves. IEEE Trans. Ind. Inform..

[17] Korzeniewska E., Kania M., Zawiślak R. (2022). Textronic Glove Translating Polish Sign Language. Sensors.

[18] Ji L., Liu J., Shimamoto S. Recognition of Japanese Sign Language by Sensor-Based Data Glove Employing Machine Learning. Proceedings of the 2022 IEEE 4th Global Conference on Life Sciences and Technologies (LifeTech).

[19] Lee M., Bae J. (2022). Real-Time Gesture Recognition in the View of Repeating Characteristics of Sign Languages. IEEE Trans. Ind. Inform..

[20] Sharma S., Singh S. (2021). Vision-based hand gesture recognition using deep learning for the interpretation of sign language. Expert Syst. Appl..

[21] Dipietro L., Sabatini A.M., Dario P. (2008). A Survey of Glove-Based Systems and Their Applications. IEEE Trans. Syst. Man, Cybern. Part C.

[22] Ramos O., Múnera M., Moazen M., Wurdemann H., Cifuentes C.A. (2022). Assessment of Soft Actuators for Hand Exoskeletons: Pleated Textile Actuators and Fiber-Reinforced Silicone Actuators. Front. Bioeng. Biotechnol..

[23] Kim G., Vu C.C., Kim J. (2020). Single-Layer Pressure Textile Sensors with Woven Conductive Yarn Circuit. Appl. Sci..

[24] Nakajima T., Asami Y., Endo Y., Tada M., Ogihara N. (2022). Prediction of anatomically and biomechanically feasible precision grip posture of the human hand based on minimization of muscle effort. Sci. Rep..

[25] Leonardis D., Barsotti M., Loconsole C., Solazzi M., Troncossi M., Mazzotti C., Castelli V.P., Procopio C., Lamola G., Chisari C. (2015). An EMG-Controlled Robotic Hand Exoskeleton for Bilateral Rehabilitation. IEEE Trans. Haptics.

[26] Erol A., Bebis G., Nicolescu M., Boyle R., Twombly X. (2007). Vision-based hand pose estimation: A review. Comput. Vis. Image Underst..

[27] Mohamed N., Mustafa M.B., Jomhari N. (2021). A Review of the Hand Gesture Recognition System: Current Progress and Future Directions. IEEE Access.

[28] Nishiyama M., Watanabe K. (2009). Wearable Sensing Glove With Embedded Hetero-Core Fiber-Optic Nerves for Unconstrained Hand Motion Capture. IEEE Trans. Instrum. Meas..

[29] Dipietro L., Sabatini A.M., Dario P. (2003). Evaluation of an instrumented glove for hand-movement acquisition. J. Rehabil. Res. Dev..

[30] Chang H.T., Chang J.Y. (2020). Sensor Glove Based on Novel Inertial Sensor Fusion Control Algorithm for 3-D Real-Time Hand Gestures Measurements. IEEE Trans. Ind. Electron..

[31] Kortier H.G., Sluiter V.I., Roetenberg D., Veltink P.H. (2013). Assessment of hand kinematics using inertial and magnetic sensors. J. Neuroeng. Rehabil..

[32] Lin B.S., Lee I.J., Yang S.Y., Lo Y.C., Lee J., Chen J.L. (2018). Design of an Inertial-Sensor-Based Data Glove for Hand Function Evaluation. Sensors.

[33] Hsiao P.C., Yang S.Y., Lin B.S., Lee I.J., Chou W. Data glove embedded with 9-axis IMU and force sensing sensors for evaluation of hand function. Proceedings of the 2015 37th Annual International Conference of the IEEE Engineering in Medicine and Biology Society (EMBC).

[34] Saggio G., Bocchetti S., Pinto C.A., Orengo G., Giannini F. A novel application method for wearable bend sensors. Proceedings of the 2009 2nd International Symposium on Applied Sciences in Biomedical and Communication Technologies.

[35] Polygerinos P., Wang Z., Galloway K.C., Wood R.J., Walsh C.J. (2015). Soft robotic glove for combined assistance and at-home rehabilitation. Robot. Auton. Syst..

[36] Yap H.K., Ang B.W.K., Lim J.H., Goh J.C.H., Yeow C.H. A fabric-regulated soft robotic glove with user intent detection using EMG and RFID for hand assistive application. Proceedings of the 2016 IEEE International Conference on Robotics and Automation (ICRA).

[37] Stoppa M., Chiolerio A. (2014). Wearable Electronics and Smart Textiles: A Critical Review. Sensors.

[38] Huang C.T., Shen C.L., Tang C.F., Chang S.H. (2008). A wearable yarn-based piezo-resistive sensor. Sensors Actuators Phys..

[39] Aumüller G., Aust G., Conrad A., Engele J., Kirsch J. (2017). Duale Reihe Anatomie.

[40] Witte H., Schilling C. (2017). The concept of biomechatronic systems as a means to support the development of biosensors. Int. J. Biosens. Bioelectron..

[41] (VDI), V.D.I. (2004). Entwicklungsmethodik für Mechatronische Systeme (VDI 2206): Design Methodology for Mechatronic Systems.

[42] Durlach N.I., Mavor A.S., National Research Council (1995). Virtual Reality: Scientific and Technological Challenges.

[43] Li Y., Chen X., Zhang X., Wang K., Wang Z.J. (2012). A Sign-Component-Based Framework for Chinese Sign Language Recognition Using Accelerometer and sEMG Data. IEEE Trans. Biomed. Eng..

[44] Weber W. (2019). Industrieroboter: Methoden der Steuerung und Regelung.

[45] Park Y., Lee J., Bae J. (2015). Development of a Wearable Sensing Glove for Measuring the Motion of Fingers Using Linear Potentiometers and Flexible Wires. IEEE Trans. Ind. Inform..

[46] Tilley A., Associates H. (2002). The Measure of Man and Woman: Human Factors in Design.

[47] Burfeind H. (2004). Zur Biomechanik des Fingers unter Berücksichtigung der Krümmungsinkongruenz der Gelenkflächen.

[48] de Monsabert B.G., Visser J.M.A., Vigouroux L., Van der Helm F.C.T., Veeger H.E.J. (2014). Comparison of three local frame definitions for the kinematic analysis of the fingers and the wrist. J. Biomech..

[49] Chao E., An K., Conney W., Linscheid R. (1989). Biomechanics of the Hand: A Basic Research Study.

[50] (2021). Bionic Gloves | ReliefGrip™ Golf Gloves. https://www.bionicgloves.com/reliefgrip?quantity=1&custcol3=14.

[51] Malvezzi M., Gioioso G., Salvietti G., Prattichizzo D. (2015). SynGrasp: A MATLAB Toolbox for Underactuated and Compliant Hands. IEEE Robot. Autom. Mag..

[52] Hill J. (2020). Do deaf communities actually want sign language gloves?. Nat. Electron..

[53] Novak D., Riener R. (2015). A survey of sensor fusion methods in wearable robotics. Robot. Auton. Syst..

[54] Barsoum E. (2016). Articulated Hand Pose Estimation Review. CoRR.

[55] Dong W., Yang L., Gravina R., Fortino G. (2022). Soft Wrist-Worn Multi-Functional Sensor Array for Real-Time Hand Gesture Recognition. IEEE Sensors J..

[56] Jiang S., Li L., Xu H., Xu J., Gu G., Shull P.B. (2020). Stretchable e-Skin Patch for Gesture Recognition on the Back of the Hand. IEEE Trans. Ind. Electron..

[57] Li L., Jiang S., Shull P.B., Gu G. (2018). SkinGest: Artificial skin for gesture recognition via filmy stretchable strain sensors. Adv. Robot..

[58] Takada R., Kadomoto J., Shizuki B. A Sensing Technique for Data Glove Using Conductive Fiber. Proceedings of the Extended Abstracts of the 2019 CHI Conference on Human Factors in Computing Systems.

